# SiO_2_ Stabilized Magnetic Nanoparticles as a Highly Effective Catalyst for the Degradation of Basic Fuchsin in Industrial Dye Wastewaters

**DOI:** 10.3390/molecules23102573

**Published:** 2018-10-09

**Authors:** Jingheng Ning, Min Wang, Xin Luo, Qiongcan Hu, Rong Hou, Weiwei Chen, Donger Chen, Jianhui Wang, Jun Liu

**Affiliations:** 1 School of Chemistry and Biological Engineering, Changsha University of Science & Technology, Changsha 410110, China; wang_min1993@126.com (M.W.); luoxin_gl@126.com (X.L.); huqiongcan@126.com (Q.H.); hourong0406@163.com (R.H.); chenweiwei_2012@126.com (W.C.); chendonger9@163.com (D.C.); 2 Hunan Key Laboratory of Biomedical Nanomaterials and Devices, School of Life Sciences and Chemistry, Hunan University of Technology, Zhuzhou 412007, China; junliu@hut.edu.cn

**Keywords:** Fe_3_O_4_/SiO_2_ nanoparticles, Fenton reaction, basic fuchsin, H_2_O_2_, catalytic degradation

## Abstract

Catalytic degradation of organic pollutants by nanomaterials is an effective way for environmental remediation. The Fenton reaction involving H_2_O_2_ oxidation catalysed by Fe^3+^ is an advisable way for wastewater degradation. Herein, Fe_3_O_4_/SiO_2_ core-shell nanoparticles were prepared as catalyst by coprecipitation and sol-gel methods, and this catalyst is used for degradation of fuchsin in wastewater by H_2_O_2_. The Fenton reaction between H_2_O_2_ and Fe_3_O_4_ is proposed to explain the catalytic performance. The coating of SiO_2_ on Fe_3_O_4_ nanoparticles could dramatically stabilize the Fe_3_O_4_ in aqueous solution and prevent their oxidation. More importantly, the magnetic property of Fe_3_O_4_ nanoparticles endows them with good recyclability. Thus, due to the outstanding catalytic results, almost 100% removal degradation was achieved within 5 min over a wide pH value range at room temperature, which is better than that without catalysts. Temperature is a positive factor for improving the degradation rate, but room temperature is selected as the best temperature for economic and energy savings reasons, because more than 98% of fuchsins can still be degraded at room temperature. Moreover, these Fe_3_O_4_/SiO_2_ core-shell nanoparticles exhibit excellent magnetic recyclability and stable properties after repeated utilization. Therefore, these as-presented Fe_3_O_4_/SiO_2_ core-shell nanoparticles with low-cost and high performance are expected to be applied in practical industry wastewater degradation.

## 1. Introduction

Dye wastewater is one of the most serious sources of water pollution, as the chemical compounds in dye wastewater have many characteristics such as high organic matter content, complex composition, change of pH value and biochemical activity. Moreover, such colored effluents are putatively toxic and carcinogenic to humankind, aquatic animals and plants [1,2]. Basic fuchsin is a common triphenylmethane dye, widely used in cotton, synthetic fiber, paper, leather, printing and dyeing industrial production, but it is also a poisonous dye, its ingestion may cause gastrointestinal irritation with nausea, vomiting, and diarrhea, and the inhalation of the dye causes irritation to the respiratory tract [3]. Many techniques, including physical adsorption [4], electrochemical oxidation [5,6], biodegradation [7], catalytic wet oxidation [8], and electro-Fenton reagent oxidation [9] have been adopted for the removal of dye pollutants, but traditional dye wastewater treatment technology has some shortcomings. For example, electro-Fenton oxidation easily causes secondary pollution, and requires an acidic environment [10,11,12], and the cost of the ozone oxidation method is extremely high [13,14]. Therefore, for industrial dye wastewater degradation, especially the basic fuchsin, developing a low cost, high efficiency and reliable dye wastewater treatment technology has been particularly important. Catalytic degradation of basic fuchsin without secondary pollution at room temperature is an advisable way to achieve environmental purification.

Recent studies of basic fuchsin degradation are mainly focused on the adsorption method [15,16,17], but few literatures are focused on its direct catalytic degradation [18]. The catalytic oxidation by H_2_O_2_ based on semiconductors is an advisable method for degradation of dye wastewaters, because of the high catalytic performance and low environmental contamination. Recently, the Fenton reaction between H_2_O_2_ and Fe^3+^ is found to be a significant way in improving the oxidative activity of H_2_O_2_. Thus, magnetic nanoparticles (MNPs), such as Fe_3_O_4_ and Fe_2_O_3_, have receive wide and increasing attention as new adsorbents [19,20] and catalysts [21,22,23,24,25,26], due to their advantages such as high specific surface area, magnetic separation and recyclability and superior catalytic properties in the Fenton reaction. However, as we all know, Fe_3_O_4_ is a magnetic material, so the particles easily group together on the nanoscale. Thus, Fe_3_O_4_ nanoparticles are likely to agglomerate hampering the further catalytic reaction. Moreover, Fe_3_O_4_ nanoparticles are also unstable in air, because Fe^2+^ is easily oxidized in air. Thus, a surface coating is an excellent way for maintaining the good stability and dispersibility of Fe_3_O_4_ nanoparticles. Recently, a SiO_2_ coating is found to be a good method for improving the stability and dispersibility of semiconductors. In addition, the electrical property (positive or negative) of the semiconductor surface could be also changed after SiO_2_ coating. 

**Figure 1:**
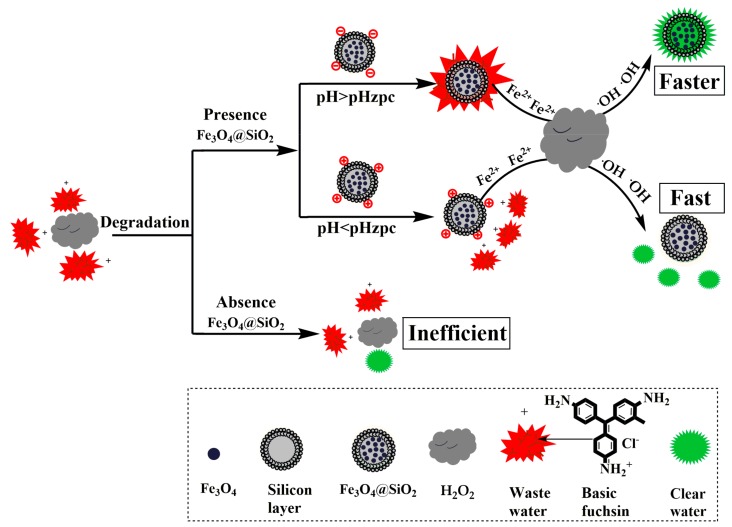
Scheme for the degradation of basic fuchsin oxidized by hydrogen peroxide and catalyzed by magnetic nanobeads.

Herein, in this paper, magnetic nanobeads/SiO_2_ composites with good dispersion in aqueous solution are prepared by a simple method. The effect of Fe_3_O_4_@SiO_2_ as a catalyst on the degradation of basic fuchsin by hydrogen peroxide is investigated (Figure 1). Moreover, many factors, such as pH value, reaction temperature and concentration of H_2_O_2_/catalyst or basic fuchsin, affecting this catalytic process are carefully investigated. In addition, the recyclability of this Fe_3_O_4_@SiO_2_ catalyst is also discussed. By using this Fe_3_O_4_@SiO_2_ catalyst, we expect that the degradation rate of basic fuchsin could be much higher than that without catalyst, and this catalyst could possess high stability, high catalytic efficiency, and good regeneration properties.

## 2. Results and Discussion 

### 2.1. Performance and Structural Characterization of Magnetic Nanoparticles

#### 2.1.1. XRD Characterization

**Figure 2:**
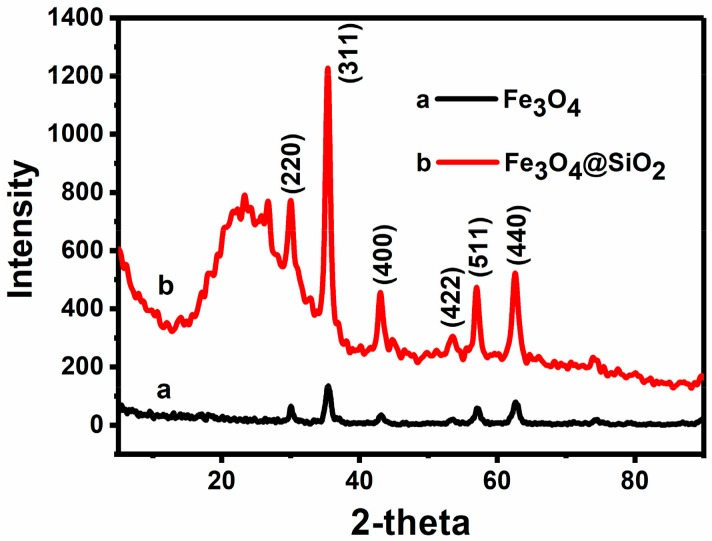
XRD of Fe_3_O_4_ MNPs and Fe_3_O_4_/SiO_2_ MNPs.

The crystalline structure of the MNPs was investigated by X-ray diffraction (XRD). As shown in Figure 2, diffraction peaks are located at 30.1°, 35.5°, 43.1°, 53.4°, 57.0° and 62.7°, respectively (line a), which correspond to the (220), (311), (400), (422), (511) and (400) crystal planes of magnetite. This is in good agreement with the data for pure cubic Fe_3_O_4_, as reported in the JCPDS card (No. 88–315, α = 8.375). This result indicates that the Fe_3_O_4_ nanoparticles are obtained, and Fe_3_O_4_ nanoparticles are successfully added to the hollow silica microspheres. Moreover, the broad peak at 22°–30° in Figure 2 could be attributed to the silica (line b), which indicates that the silica is successfully coated on the surface of the nanoparticles [27]. More importantly, from the XRD spectra of Fe_3_O_4_ nanoparticles in Figure 2, the size of Fe_3_O_4_ particles can be estimated to be 11 nm through the Scherrer formula [28,29].

#### 2.1.2. FT-IR Characterization

**Figure 3:**
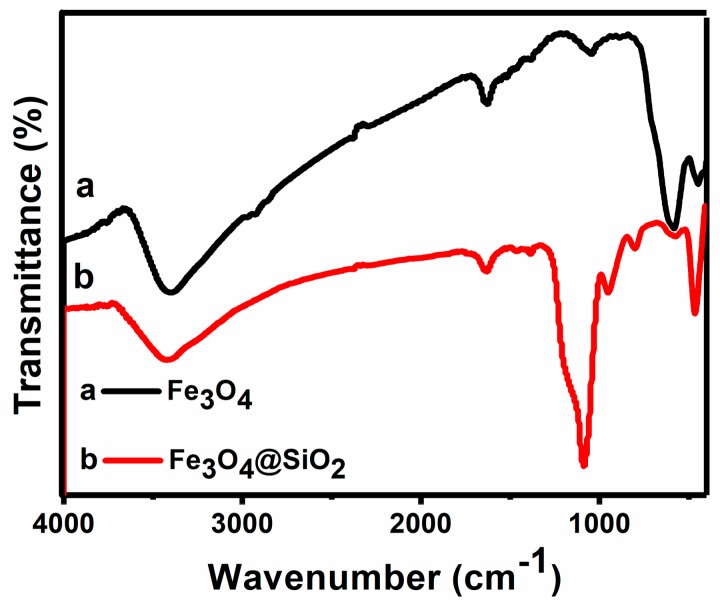
The FTIR spectra of Fe_3_O_4_ MNPs and Fe_3_O_4_/SiO_2_ MNPs.

The FTIR spectra of bare Fe_3_O_4_ MNPs and Fe_3_O_4_/SiO_2_ MNPs are presented in Figure 3 and Table 1. It is shown that the characteristic absorption bands of Fe-O bonds in the tetrahedral sites of bare MNPs were located at 582 cm^−1^. The broad band at 3300–3500 cm^−1^ is ascribed to -OH stretching vibrations. Compared with the bare MNPs, the existence of the characteristic Si-O-Si stretching at 1090 and 800 cm^−1^ on Fe_3_O_4_/SiO_2_ MNPs are evidenced to confirm the formation of the silica shell. In the spectra of Fe_3_O_4_/SiO_2_ MNPs, other characteristic absorption bands such as Si-OH stretching, Si-O bending and Si-O-Si bending are shown at 944, 800 and 464 cm^−1^, respectively. The characteristic Fe-O peak of bare MNPs at 582 cm^−1^ is shifted to 570 cm^−1^ in the spectrum of Fe_3_O_4_/SiO_2_ MNPs. Undoubtedly, it can be said that the silica shell is linked to the surface of the MNPs by Fe-O-Si chemical bond. Therefore, the above results indicate that the MNPs are successfully coated by silica. In the spectrum of Fe_3_O_4_/SiO_2_ MNPs, a short band instead of a broad band is observed at 3450 cm^−1^, which indicates the presence of O-H groups on the surface of the particles [30,31,32].

#### 2.1.3. SEM and TEM Characterization

**Figure 4:**
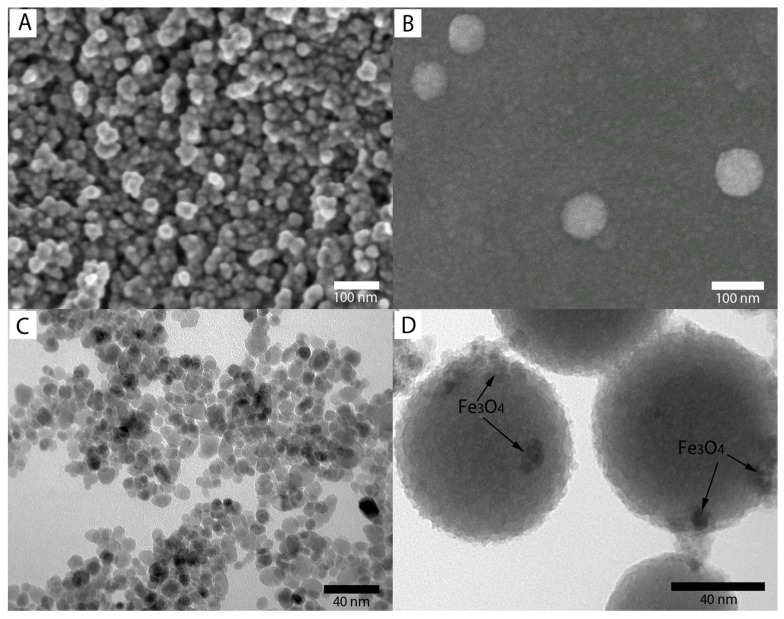
SEM images of Fe_3_O_4_ MNPs (**A**) and Fe_3_O_4_@SiO_2_ MNPs (**B**); TEM images of Fe_3_O_4_ (**C**) and Fe_3_O_4_@SiO_2_ (**D**).

**Figure 5:**
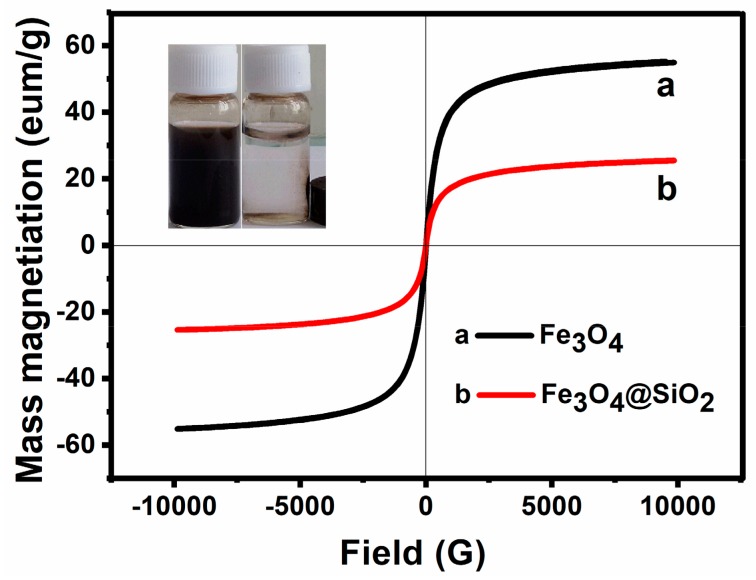
VSM characterization of Fe_3_O_4_ MNPs and Fe_3_O_4_/SiO_2_ MNPs.

SEM images (A and B) and TEM images (C and D) of Fe_3_O_4_ and Fe_3_O_4_/SiO_2_ nanoparticles are presented in Figure 4A–D, respectively. As shown in Figure 4A, the SEM image indicates that the morphology of the Fe_3_O_4_ nanoparticles is roughly spherical-shaped. Moreover, the nanoparticles show an average size of 12 nm with a narrow size distribution. This result is consistent with the estimation from XRD results (d_XRD_ = 11 nm). After coating with SiO_2_, the Fe_3_O_4_@SiO_2_ NPs (Figure 4B) exhibited a spherical shape and had a smooth surface. Compared with bare Fe_3_O_4_ NPs, the Fe_3_O_4_@SiO_2_ NPs had no apparent aggregation with an average diameter of about 70 nm. TEM images of Fe_3_O_4_ and Fe_3_O_4_/SiO_2_ nanoparticles are shown in Figure 4C,D, respectively. Figure 5C shows that the morphology of the Fe_3_O_4_ nanoparticles has a good dispersion with a narrow size distribution, and it is consistent with the SEM results. The TEM image of the Fe_3_O_4_/SiO_2_ nanoparticles is shown in Figure 4D, where the nanoparticles present spherical shapes, and they agree with the SEM results. 

#### 2.1.4. Magnetism Characterization

The magnetic properties of Fe_3_O_4_ NPs and Fe_3_O_4_@SiO_2_ NPs were studied by VSM at room temperature. As illustrated in Figure 5, the saturation magnetization of Fe_3_O_4_ NPs and Fe_3_O_4_@SiO_2_ NPs are 53.77 and 25.51 emu·g^−1^, respectively. Because of the shielding effect of nonmagnetic coatings, the magnetic response obviously decreased after coating with silica. Moreover, both of these two samples present no obvious coercive force. This means that they are paramagnetic materials. However, after coating, the modified magnetic samples still displayed a high saturation intensity (25.51 emu·g^−1^), which facilitates an easy and quick separation of Fe_3_O_4_@SiO_2_ NPs from the suspension with an external magnet (inset Figure).

### 2.2. Research of Catalytic Performance

**Figure 6:**
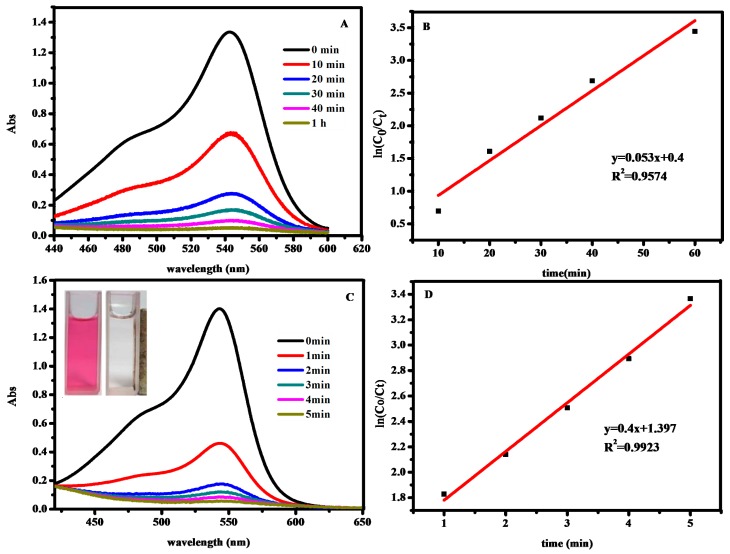
Time-dependent UV-Vis spectral changes of basic fuchsin (**A**); ln (C_0_/C_t_) and time diagram of H_2_O_2_ degradation of basic fuchsin (**B**); Time-dependent UV-Vis spectral changes of basic fuchsin with Fe_3_O_4_@SiO_2_ MNPs(C); ln (C_0_/C_t_) and time diagram of H_2_O_2_ degradation of basic fuchsin with Fe_3_O_4_@SiO_2_ MNPs(D). The inset of UV-Vis spectra shows the color of dye solution before (right) and after (left) oxidation.

A typical UV-Vis spectra of dyes’ evolution without catalytic oxidation after addition of H_2_O_2_ is shown in Figure 6A, and the reaction is complete within 1 h for basic fuchsin. These reactions follow the pseudo-first-order kinetics formula ln(C_0_/C_t_) = kt [33,34], where C_t_ is the concentration of dye at time t, C_0_ is the initial concentration of dye solution, and the slope k is the apparent work rate. ln (C_0_/C_t_) was linear with time, R^2^ = 0.9574, which can be seen from Figure 6B. The calculated rate constant for degrading basic fuchsin without Fe_3_O_4_@SiO_2_ nanoparticles was 0.053 min^−1^. Meanwhile, the catalytic oxidation reaction is completed within 5 min (Figure 6C). It is also followed with pseudo-first-order kinetic, the linear relationship between ln (C_0_/C_t_) and reaction time are observed, the rate constant is 0.4 min^−1^, and R^2^ is 0.9923 (Figure 6D). These work rates in the presence of Fe_3_O_4_@SiO_2_ nanoparticles was much higher than that the reaction without catalyst. The insets show that the dye solution become almost colorless (right side) where the nanohybrids accumulate, while there is no obvious change in color on the left side. This result clearly confirms that the Fe_3_O_4_@SiO_2_ MNPs possess magnetic-catalytic dual functionalities.

#### 2.2.1. Effect of Hydrogen Peroxide Concentration on the Degradation Reaction

**Figure 7:**
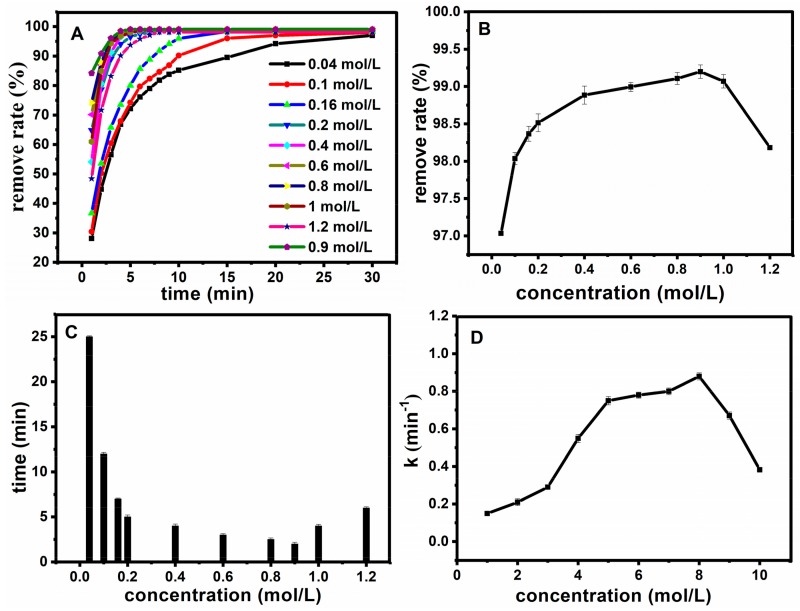
Effect of H_2_O_2_ concentration on oxidation rate (**A**); The relationship between the concentration of H_2_O_2_ and the removal degree after 30 min (**B**); the relationship between the hydrogen peroxide concentration and time when the removal degree reached 98% (**C**); the relationship between the concentration of hydrogen peroxide and the work rate (**D**).

**Figure 8:**
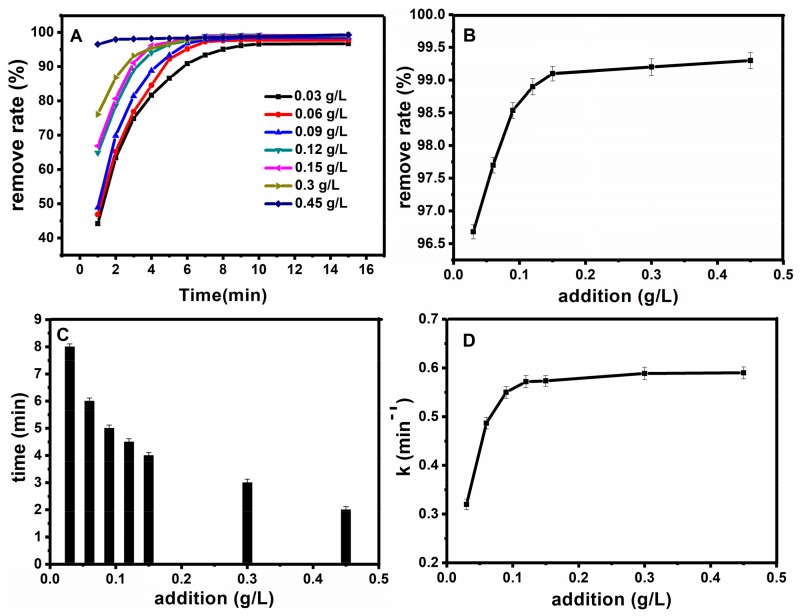
Effect of catalyst dosage on oxidation rate (**A**); the relationship between the catalyst dosage and the removal degree after half an hour (**B**); the relationship between the catalyst dosage and time when the removal degree reached 98% (**C**); the relationship between the catalyst dosage and the work rate (**D**).

After determining the catalytic properties of MNPs, the conditions for the catalytic degradation were optimized. Firstly, for the optimization of hydrogen peroxide concentration, different concentrations of hydrogen peroxide were added for Fe_3_O_4_@SiO_2_ catalytic degradation of basic fuchsin by fixing other conditions as follows: the temperature T = 25 °C, C_FB_ = 10 mg·L^−1^, C_Fe3O4@SiO2_ = 0.12 g·L^−1^ and pH = 7. Results are shown in Figure 7A. Diagrams of the influence of hydrogen peroxide concentrations on removal degree are presented in Figure 8B, from which we can see that suitable concentrations of hydrogen peroxide could decolorize more than 95% of the fuchsin basic within 30 min. In order to show the effect of hydrogen peroxide concentration on work rate, the relationship between different hydrogen peroxide concentrations and reaction time and the rate were shown at Figure 7B–D. The removal degree increased with the increase of concentration in the range of 0.04 to 0.9 mol·L^−1^, and decreased once the concentration was above 1 mol·L^−1^. Figure 7C shows the relationship between the rate of hydrogen peroxide concentrations and time, when the removal degree reached 98%. Figure 7D reveals the relationship between the concentration of hydrogen peroxide and the work rate. With the increase of concentration, the reaction time decreased and the work rate increased gradually in the range of 0.04 to 0.9 mol·L^−1^. However, opposite results were observed after more than 1 mol·L^−1^. This phenomenon may be attributed to the fact that hydrogen peroxide could produce ·OH for the degradation of fuchsin. Basically, a low concentration of hydrogen peroxide could cause less ·OH generation; while the amount of ·OH are increasing with the increase of hydrogen peroxide concentration, which increased the degradation rate of fuchsin basic. However, when the hydrogen peroxide concentration increased to a certain extent, excess H_2_O_2_ could also act as a ·OH scavenger, and HOO· was formed by excess ·OH (chemical Equation (1)). At the same time, the adverse work rate is increased, and as a consequence, the degradation rate of fuchsin basic decreased. Thus, 0.9 mol·L^−1^ was chosen as the optimum dosage of H_2_O_2_.

H_2_O_2_ + ·OH → H_2_O + HOO (1)

#### 2.2.2. Influence of Catalyst Dosage on the Degradation Reaction

After determining the best concentration of hydrogen peroxide, the dosage of magnetic nanobeads was optimized. Firstly, for the optimization of catalyst dosage, different catalyst dosages were investigated for the Fe_3_O_4_@SiO_2_ catalytic degradation of basic fuchsin correspondingly, while the other conditions were fixed as follows: the temperature T = 25 °C, C_H2O2_ = 0.9 mol·L^−1^, C_FB_ = 10 mg·L^−1^, pH = 7. Figure 8A shows the influence of catalyst dosage on removal degree, from which we can see that a certain catalyst dosage could decolorize more than 95% of the fuchsin basic within 10 min. Figure 8B shows the relationship between the catalyst dosage and the removal degree, and as shown in this figure, the removal degree increased with the increase of catalyst dosage. Figure 8C shows the relationship between catalyst dosage and time, when the removal degree reached 98%. The degree of fuchsin basic removal increased gradually with the increase of the concentration of Fe_3_O_4_@SiO_2_. Moreover, the decolorization time decreased when it reached more than 95%. On the one hand, increasing the amount of Fe_3_O_4_@SiO_2_ provides greater reaction area to the reaction. On the other hand, more Fe_3_O_4_@SiO_2_, by which fuchsin basic was adsorbed, contributed to increasing the oxidation rate of fuchsin greatly. But this “increasing” trend didn’t continue when the Fe_3_O_4_@SiO_2_ concentration was greater than 0.15 g·L^−1^, which is probably due to the consumption of H_2_O_2_, and the removal degree of basic fuchsin cannot be improved by further addition of Fe_3_O_4_@SiO_2_, because the absorption equilibrium is reached. Figure 8D revealed the relationship between the catalyst dosage and the work rate. With the increase of concentration, the reaction time decreased while the work rate increased gradually. We can generalize a conclusion from this figure that work rate could increase when the Fe_3_O_4_@SiO_2_ concentration increased from 0.06 g·L^−1^ to 0.15 g·L^−1^. However, the work rate is increasing slowly in the Fe_3_O_4_@SiO_2_ concentration range of 0.15 g·L^−1^ to 0.45 g·L^−1^. This result can be interpreted as a trend that the adsorption of basic fuchsin will increase significantly with the increase of Fe_3_O_4_@SiO_2_. Thus 0.15 g·L^−1^ is chosen as the optimum dosage of Fe_3_O_4_@SiO_2_.

#### 2.2.3. Effect of Initial Concentration of Basic Fuchsin on the Degradation Reaction

**Figure 9:**
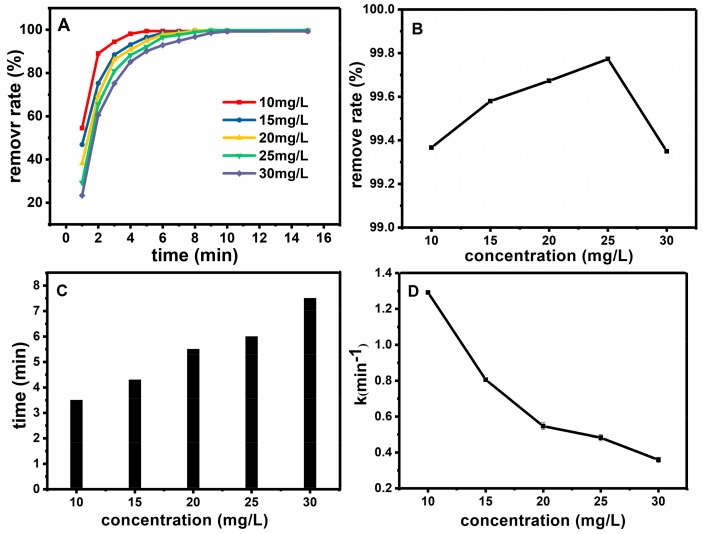
Effect of initial concentration of basic fuchsin on oxidation rate (**A**); The relationship between the initial concentration of basic fuchsin and the removal degree after half an hour (**B**); The relationship between initial concentration of basic fuchsin and time when the removal degree reached 98% (**C**); the relationship between initial concentration of basic fuchsin and the work rate (**D**).

The effect of the initial concentration of basic fuchsin on the degradation efficiency was also investigated. These experiments were carried out under the condition of room temperature, C_H2O2_ = 0.9 mol·L^−1^, C_Fe3O4@SiO2_ = 0.15 g·L^−1^, pH = 7. Figure 9A reflects the influence of initial concentration of basic fuchsin on removal degree. With different initial concentration of basic fuchsin, the removal degree can reach more than 95% within 10 min. Figure 9B shows the relationship between the initial concentration of basic fuchsin and the removal degree after half an hour. Figure 9C illustrates the relationship between the initial concentration of basic fuchsin and the time when the removal degree reached 98%. Figure 9D shows the relationship between the initial concentration of basic fuchsin and the work rate. As a result, with the increase of initial concentration of basic fuchsin, the removal degree gradually decreased, and more time is needed for reaching 98% removal. Thus 10 mg·L^−1^ is chosen as the optimum value of the initial concentration of basic fuchsin. 

#### 2.2.4. Temperature Effect on the Degradation Reaction

**Figure 10:**
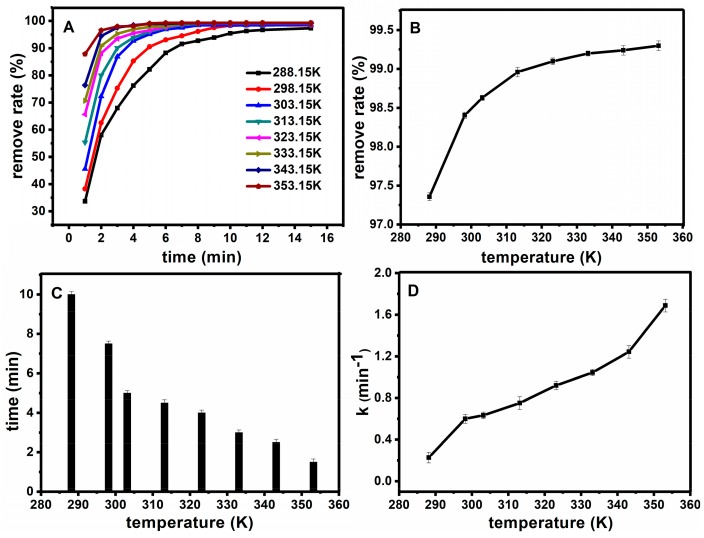
Effect of temperature on oxidation rate (**A**); The relationship between temperature and the removal degree after half an hour (**B**); The relationship between temperature and time when the removal degree reached 98% (**C**); the relationship between temperature and the work rate (**D**).

Under the optimal catalytic conditions, the temperature conditions were further selected, and the experiments were carried out at C_FB_ = 10 mg·L^−1^, C_H2O2_ = 0.9 mol·L^−1^, C_Fe3O4@SiO2_ = 0.15 g·L^−1^, pH = 7. Figure 10A shows the effect of temperature on oxidation rate, the fuchsin basic removal degree can reach more than 95% in 15 min at different temperatures. Figure 10B shows the relationship between temperature and the removal degree after 30 min. Figure 10C illustrates the relationship between temperature and time when the removal degree reached 98%. Figure 10D shows the relationship between temperature and the work rate. With the increase of temperature, the removal degree gradually increased. When the reaction time changed from 15 min to 2 min, the degradation rate increased because the speed of molecular motion was promoted, which leads to an increase in the opportunity for collisions between the basic fuchsin and Fe_3_O_4_@SiO_2_ on the surface with the increase of temperature. Nevertheless, a higher reaction temperature can provide more energy for reactant molecules to overcome the activation energy, and the generation of free radicals could multiply with the increase of reaction temperature. At the same time, Fe_3_O_4_@SiO_2_ still has a high activity when it is heated to 80 °C, which means that the catalyst is stable. 

#### 2.2.5. Effect of pH on the Mechanism of Catalysis

**Figure 11:**
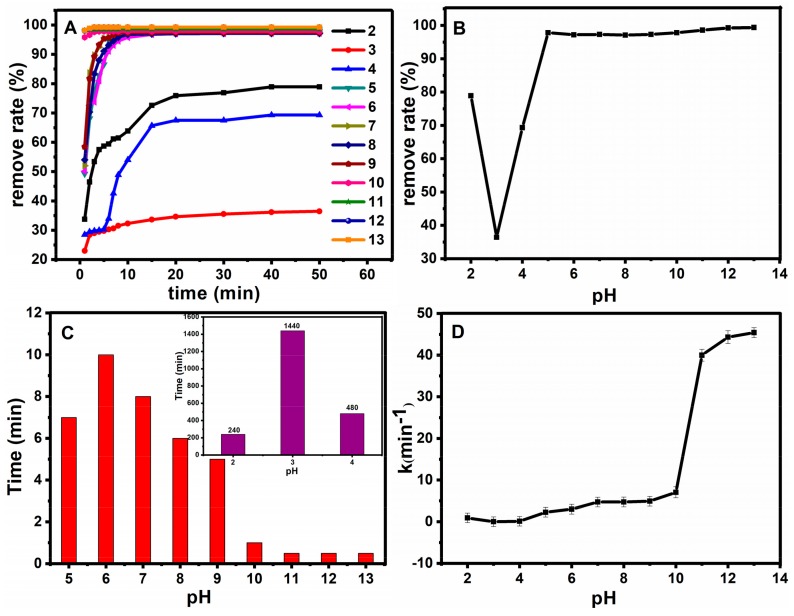
Effect of pH on oxidation rate (**A**); The relationship between pH and the removal degree after half an hour (**B**); The relationship between pH and time when the removal degree reached 98% (**C**); the relationship between pH and the work rate (**D**).

The pH value also plays a key role in the procedure. An appropriate pH value can improve the catalysis efficiency, and it also reduces interferences from the sample matrix. Therefore, the pH of the model solution was adjusted in the range of 2–13 at room temperature. The results are shown in Figure 11. Figure 11A shows the effect of pH on oxidation rate. Figure 11B–D show the relationship between pH and the removal degree after half an hour, the relationship between pH and time when the removal degree reached 98%, and the relationship between pH and the work rate, respectively. The degradation rate of basic fuchsin decreased with the increase of pH when the pH < 3. However, under the environment of pH > 3, basic fuchsin removal degree gradually increased with the increase of pH value. Thus, the reaction time was reduced significantly. It can be seen that the Fe_3_O_4_@SiO_2_ still maintains good activity under strong acid and strong base conditions, which further illustrates the stability of the catalyst.

High hydrogen ion concentration will promote the reaction of oxygen consumption (chemical equation (2)) under low pH conditions, which leads to the decrease of active sites for formation of H_2_O_2_. Because the surface zero charge point of Fe_3_O_4_@SiO_2_ (pHzpc) is about 2.8 [7] to 3.4 [19], when pH < pHzpc, a relatively small amount of dissolved ferrous ion forms the Fenton system with hydrogen peroxide [35]. This can promote the decomposition of hydrogen peroxide, and improve the work rate. However, owning to the positively-charged surface of Fe_3_O_4_@SiO_2_, and it is repelled by positive basic fuchsin. Thus, the adsorption ratio of basic fuchsin subsequently decreased. The reaction between ·OH (produced from the Fenton system) and basic fuchsin is not occurring on the surface of Fe_3_O_4_@SiO_2_. This causes a decrease of the reaction area, and the reaction process is shown in chemical Equations (3) and (4). On the other hand, the Fenton reaction system is also accompanied by other reactions, which are not conducive to the existence of ·OH, as shown in chemical Equations (5) and (6). Moreover, due to the neutralization reaction between acid and basic fuchsin, the reaction and removal degree is reduced with the decrease of hydrogen ion concentration under strong acidic conditions. The work rate could decrease with the increase of pH when pH < pHzpc, which is consistent with the experimental results.

H_2_O_2_ + 2H^+^ + 2e^−^ → 2H_2_O (2)

H_2_O_2_ + Fe^2+^ → ·OH + OH^−^ + Fe^3+^ (3)

Fe^3+^ + H_2_O_2_ → Fe^2+^ + HO_2_· + H^+^ (4)

·OH + Fe^2+^ → OH^−^ + Fe^3+^ (5)

·OH + H_2_O_2_ → H_2_O + HO_2_ (6)

Secondly, the surface of Fe_3_O_4_@SiO_2_ is negatively charged when the pH > pHzpc, it can attract the positively charged basic fuchsin, leading to the increase of adsorption rate of fuchsin basic. On the other hand, the reaction between ·OH and fuchsin basic happened on the surface of Fe_3_O_4_@SiO_2_, which provides a large area for reactions. The action of the two effects can greatly improve the work rate. Consequently, the work rate increases with the increase of pH when pH > pHzpc. Therefore, basic fuchsin molecules are more easily adsorbed and degraded on the surface of Fe_3_O_4_@SiO_2_ in an alkaline environment, and this result is also consistent with the experimental data.

### 2.3. Catalytic Recyclability of Fe_3_O_4_@SiO_2_ NPs

**Figure 12:**
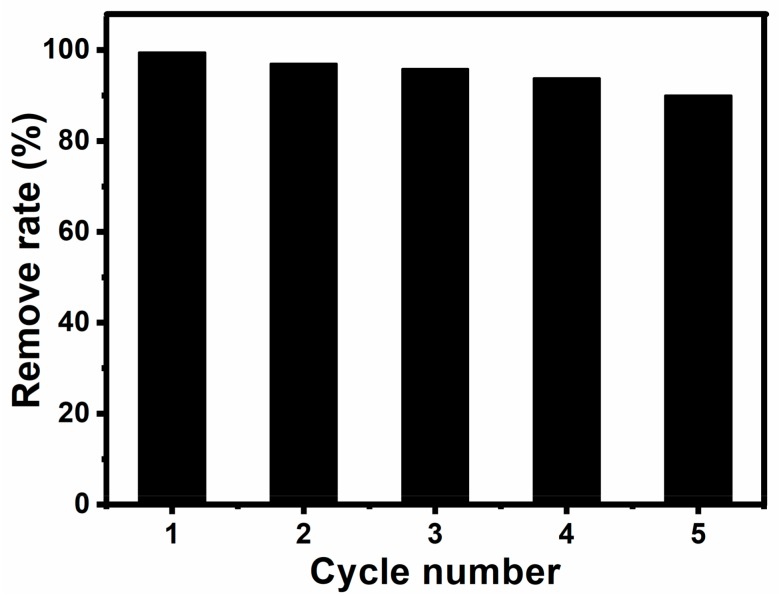
Reusability of Fe_3_O_4_@SiO_2_ MNPs as a catalyst for the oxidation of basic fuchsia at the presence of H_2_O_2_.

We demonstrated a facile, effective route to fabricate Fe_3_O_4_@SiO_2_ nanospheres. The as-prepared nanohybrids can be used as a high-performance catalyst for the oxidation of basic fuchsin. MNPs are easily separated by an external magnetic field, and then washed with deionized water until the supernatant fluid was colorless. Finally, the MNPs can also dispersed in deionized water rapidly. Furthermore, Fe_3_O_4_@SiO_2_ catalysts can be recycled simply by magnetic separation for several times (Figure 12). Their catalytic activity decreases slightly after each cycle, and the oxidation efficiency on basic fuchsin was 90% after five cycles, which indicates that Fe_3_O_4_@SiO_2_ nanohybrids can be used as a convenient recyclable catalyst. These MNPs are rapid, efficient and recyclable catalysts for dye pollutants, and they can be applied for catalytic oxidation of other reductive contaminants.

## 3. Experimental Section

### 3.1. Materials and Instrumentation

Materials: The following chemicals were used in this study: tetraethoxysilane (TEOS) (XiLong Chemical, Guangzhou, China), iron (II) chloride tetrahydrate (Sinopharm Chemical Reagent Co., Ltd, Shanghai, China), iron (III) chloride hexahydrate (Sinopharm Chemical Reagent Co., Ltd.), Tetramethylammonium hydroxide (Sinopharm Chemical Reagent Co., Ltd.), basic fuchsin (Sinopharm Chemical Reagent Co., Ltd.), ammonium hydroxide (Chongqing Chuandong Chemical Group Co., Ltd, Chongqing, China), hydrogen peroxide (30%, Sinopharm Chemical Reagent Co., Ltd.), ethyl alcohol (Anhui Antell Food Co., Ltd., Hefei, China), sulfuric acid (Sinopharm Chemical Reagent Co., Ltd.), sodium hydroxide (Sinopharm Chemical Reagent Co., Ltd.). All the chemicals were of analytical reagent grade and were used as received without any further purification.

Instrumentation: Scanning electron microscope (SEM) (JEM-3010, Shimadzu Corporation, Tokyo, Japan); Transmission electron microscopy (TEM) (Hitachi-8100, Japan JEOL Corporation, Tokyo, Japan); X-ray powder diffraction (XRD) (MAX-2500, Neo-Confucianism Japanese Company, Tokyo, Japan); UV-Vis Spectrophotometer (TU-1901, Beijing Purkinje General Instrument Co., Ltd., Beijing, China); Vibrating sample magnetometer (VSM) (Lake Shore VSM 7307, Lake Shore Company, Washington, MI, USA); Infrared spectrometer (FT-IR) (Avatar 360, Thermo Nicolet Corporation, Madison, WI, USA); Ultrasonic cleaner (KQ-50B, Kunshan Ultrasonic Instruments Co., Ltd, Kunshan, China); pH meter (Kunshan Ultrasonic Instruments Co., Ltd.).

### 3.2. Methods

#### 3.2.1. Synthesis of Fe_3_O_4_

MNPs were synthesized by a chemical coprecipitation method under a molar ratio of 1:2 of Fe^2+^ salt and Fe^3+^ salt [36], as shown in Figure 2. Then, an aqueous NH_3_·H_2_O solution (25%) was added to it. After reaction at room temperature for 30 min under mechanical stirring, the Fe_3_O_4_ NPs solid precipitate was magnetically separated, thereafter washed with deionized water until the the supernatant fluid was transparent. After that, tetramethylammonium hydroxide aqueous solution (25%) was added into the Fe_3_O_4_ NPs solid precipitates, and diluted to 40 mL with deionized water after mechanical stirring for 10 min. Finally, the Fe_3_O_4_ NPs solid precipitates were treated with ultrasonic irradiation for 1 h, and a bright black magnetic Fe_3_O_4_ nanoparticles solution was obtained ultimately.

#### 3.2.2. Synthesis of Fe_3_O_4_@SiO_2_

**Figure 13:**
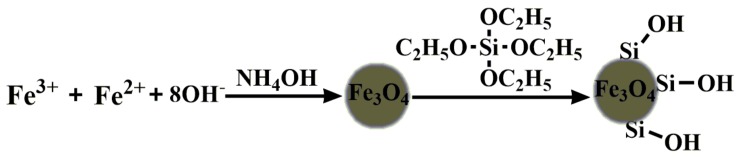
Synthetic route of MNPs.

The Fe_3_O_4_@SiO_2_ nanoparticles were synthesized by a literature method [37] with slight improvements, as shown in Figure 13. Firstly, a solution of 10 mL of distilled water, 50 mL of absolute alcohol, 5 mL of aqueous ammonia and 0.4 mL of TEOS was mechanically stirred at room temperature for 30 min, then 40 mg magnetic Fe_3_O_4_ nanoparticles solution was added into the above solution, and the mixture solution was treated with ultrasound or 30 minutes and then continuously stirred for 12 h at room temperature, Then the solid precipitates were magnetically separated. Finally, before re-dispersing in deionized water, the products were washed with deionized water until the supernatant fluid was transparent.

#### 3.2.3. Catalytic Reaction

Fe_3_O_4_@SiO_2_ nanoparticles (0.06 g·L^−1^) was injected into basic fuchsin solution (10 mg·L^−1^) in a 25 mL conical flask, then the pH was adjusted to a desired value using a pH meter. Next, 0.2 mol·L^−1^ of H_2_O_2_ was added to the above solution, and the Fe_3_O_4_@SiO_2_ nanoparticles were separated with an applied magnetic field. Finally, the basic fuchsin concentration of the supernatant fluid was determined by a UV-vis spectrophotometer, and the fuchsin removal degree was calculated. The process of magnetic nanobeads use as a catalyst for the degradation of basic fuchsin by hydrogen peroxide is presented in Figure 1 [38,39].

## 4. Conclusions

In summary, SiO_2_-stabilized Fe_3_O_4_ NPs have been synthesized for the degradation of basic fuchsin with H_2_O_2_ addition, and the SiO_2_ coating on the Fe_3_O_4_ nanoparticles could stabilize the Fe_3_O_4_ in aqueous solution and prevent its oxidation. The degradation effect is significantly higher than that of using H_2_O_2_ alone. The catalytic work rate is calculated as 0.4 min^−1^, which is higher than that without catalyst (0.053 min^−1^). Moreover, the degradation and discoloration rate of fuchsin already reached to a high level at room temperature. The optimum conditions are obtained when the C_H2O2_ = 0.9 mol·L^−1^ and C_Fe3O4@SiO2_ = 0.15 g·L^−1^. The degradation of basic fuchsin decreased with the increase of pH when the pH < 3, and the fuchsin basic removal degree gradually increased with the increased of pH when the pH > 3. Moreover, the Fe_3_O_4_@SiO_2_ catalyst can be used five times, without any obvious decrease in the degradation effect, and the catalyst can be easily separated by an external magnetic field. Finally, the catalyst remains stable in high temperature, strong acid and alkali conditions, indicating its good adaptation to a wide pH range. Thus, the as-presented Fe_3_O_4_/SiO_2_ core-shell nanoparticles with low-cost and high catalytic performance are expected to be applied in practical industry wastewater degradation.

## Figures and Tables

**Table 1:** FTIR data of Fe_3_O_4_ MNPs and Fe_3_O_4_/SiO_2_ MNPs.

Samples	Wavenumber (cm^−1^)	Attribution
Fe_3_O_4_	582 cm^−1^	Fe-O
	3300–3500 cm^−1^	-OH stretching vibration
Fe_3_O_4_/SiO_2_	570 cm^−1^	Fe-O
	1090 cm^−1^	Si-O-Si
	944 cm^−1^	Si-OH stretching vibration
	800 cm^−1^	Si-O bending vibration
	464 cm^−1^	Si-O-Si bending vibration
	3300–3500 cm^−1^	-OH stretching vibration
